# The screen for cognitive impairment in psychiatry (SCIP) as a routinely applied screening tool: pathology of acute psychiatric inpatients and cluster analysis

**DOI:** 10.1186/s12888-021-03508-4

**Published:** 2021-10-09

**Authors:** Petra Schmid, Agata Czekaj, Jürgen Frick, Tilman Steinert, Scot E. Purdon, Carmen Uhlmann

**Affiliations:** 1 Department for Psychiatry and Psychotherapy I of the University of Ulm, ZfP Suedwuerttemberg Ravensburg-Weissenau, Weingartshofer Str. 2, D-88214 Ravensburg, Weissenau Germany; 2 Alberta Hospital Edmonton and the Department of Psychiatry, University of Alberta, Edmonton, Alberta Canada

**Keywords:** Schizophrenia, Psychosis, Cognition, Improvement, Neuropsychological abnormality, Cluster

## Abstract

**Background:**

Cognitive dysfunction has been reported in acute psychiatric patients for a long time. The detection of cognitive deficits is crucial both for clinical treatment and for predicting the psychosocial functional level in the further course of the disease. The SCIP is a well-evaluated screening instrument for the examination of cognitive performance in psychiatric patients. We recently integrated the SCIP into our routine admission and discharge assessments on two inpatient wards, and we examined the cognitive profiles of patients with psychotic and affective disorders over the course of their admission.

**Methods:**

Shortly after admission, and prior to discharge, patients were routinely referred for examination with the SCIP. A total of 529 assessments were completed on admission, and 227 returned for SCIP at the time of discharge. After standardization of the test results against a normative sample, we examined the normalized test values in terms of percentages of pathological cognitive performance based on the total SCIP score, and each of the SCIP subscale scores. We conducted cluster analysis to identify cognitive subgroups within the clinical sample.

**Results:**

More than 70% of the SCIP results on admission were pathological. At discharge, improvements were observed, especially on tests with attention and speed components. Cluster analysis identified two groups. The cluster with chronic patients showed poorer results at admission, but greater improvement and reached the level of the others at discharge.

**Conclusions:**

The SCIP appears to have value in routine diagnostic assessments, and in the quantification of improvements in cognitive performance during an inpatient stay. The greatest benefit was observed in chronically ill patients with many previous stays.

**Trial registration:**

DRKS00019825 (retrospectively registered on 03.12.2019).

## Background

Neuropsychological deficits have been reported in psychosis since Kraepelin [1] and are still of high interest [2]. A meta-analysis of patients with psychotic disorders showed deficits in all cognitive areas, especially in the domains of verbal memory and processing speed [3]. It was also observed that executive functions are impaired in psychotic patients [4]. Deficits in executive function, attentional performance and memory are also described for patients with bipolar disorders [5] and for patients with depressive disorders [6, 7]. Comparing the cognitive performance of patients with psychosis and affective disorders, no differences were found [8–11]. This is in contrast to the results of Bonner-Jackson et al. [12]. The authors had examined 244 patients diagnosed with schizophrenia, other psychosis or non-psychotic depression over a period of 20 years after index treatment. At all seven points of measurement, the cognitive performance of patients with schizophrenia was significantly worse than patients with other disorders. Again, deficits in processing speed were the most common. Inconsistent findings were reported on the stability of the described deficits over the course of relatively brief inpatient treatment, with some reports of no improvement in cognitive deficits between admission and discharge [9, 13], and other reports of demonstrated improvements [10].

Cognitive deficits have considerable prognostic impact [14, 15]. It is widely accepted that the correlation between cognitive impairment and psychosocial functional level or occupational activity is significantly stronger than between clinical symptoms and functional level or occupational activity [14, 15]. The cognitive functional level proved to be a good predictor for the number and quality of social relationships [16]. Tourjman et al. [17] showed for depressed patients that the results of a cognitive screening were associated with severity of illness, self-reported cognitive dysfunction and impairment in daily life. Consequently, the assessment of cognitive performance is relevant for therapy and rehabilitation planning, but also for providing realistic job and employment perspectives [18–20].

Screening instruments are economical and easy to apply in everyday clinical practice. Several screening methods are available for the detection of cognitive disturbances in mental disorders. The Screen for Cognitive Impairment in Psychiatry (SCIP, [21]) and the Brief Assessment of Cognition in Schizophrenia (BACS, [22]) were designed for use in psychiatric samples. The SCIP requiring slightly less administration time compared to the BACS and it appears to have greater sensitivity to cognitive deficits associated with psychosis [2] compared to the established Montreal Cognitive Assessment (MoCA, [23]).

Several international studies have offered support for the utility of the SCIP as a screening tool in psychiatry applying the SCIP are already available [2, 8, 17, 24–26]. The primary emphasis of the prior reports concerned the psychometric properties of the SCIP in samples that ranged in size from *n* = 40 [17] to a high *n* = 514 [8]. Our goal was to extend these results by examination of the utility of integrating the SCIP into the routine clinical assessments undertaken on admission and discharge within a more typical high volume inpatient environment. To this end, the SCIP was added to the routine evaluations undertaken within two general psychiatric wards in 2012. Since that time we have administered the SCIP to *n* = 529 patients on admission. The aim of this study was to examine and compare the cognitive profiles of a diverse group of acute psychiatric inpatients at admission, and to assess any changes in profile that may occur between admission and discharge. Both absolute and normalized test values of performance were analyzed. Furthermore, we examined whether different clusters could be found based on the cognitive performance profiles.

## Methods

### Sample and study design

The present study is an evaluation of routinely collected clinical data that included an assessment of cognitive performance with the SCIP. Study participants were inpatients of the two acute psychiatric wards of the Clinic for Psychiatry and Psychotherapy I of the University of Ulm (Germany) at ZfP Suedwuerttemberg Ravensburg-Weissenau. Inclusion criteria were inpatient treatment, age between 18 and 65 years and ICD-10 primary diagnosis of a psychotic (F2) or an affective disorder (F3). Patients with other ICD-10 primary diagnoses, language barriers or who were not able or not willing to participate were excluded. The presence of other disorders as secondary diagnosis was not a reason for exclusion. The patients were examined at admission and again shortly before discharge. On average, admission testing was performed 10.7 days after admission (SD = 16.7) and discharge testing was performed 3.6 days (SD = 12.4) before discharge. Data analysis was performed with the approval of the responsible ethics committee of the University of Ulm (vote of August 21, 2019, no. 228–19). The study was registered at the German Register of Clinical Studies (DRKS00019825).

### Materials

#### Screen for cognitive impairment (SCIP)

The SCIP (Screen for Cognitive Impairment in Psychiatry, [21]) is a well-studied screening tool for detecting cognitive impairment in patients with psychotic or affective disorders [2, 17, 27]. The SCIP consists of five subscales: verbal learning test - immediate (VLT-I), working memory test (WMT), verbal fluency test (VFT), verbal learning test - delayed (VLT-D) and processing speed test (PST). There are three different test forms to facilitate test repetition [27] and therefore reducing learning effect. The SCIP is available as a paper-pencil test, it consists of one page and takes about 15 min to complete [2, 25–27]. Subscale scores are calculated for each of the five tests, and a total score is calculated from the sum of the subscale scores. A higher value indicates a better cognitive performance. The SCIP is available in a many languages including English, Spanish, French, Italian, Danish and German [2, 11, 21, 25–29]. With regard to its psychometric properties, the SCIP has been investigated in various validation studies in healthy and ill people. All versions showed good psychometric properties [2, 8, 25–27, 29]. However, the German version has not yet been examined in this regard.

#### Pilot: validation of the German version of the SCIP

Initially we undertook a pilot investigation of the SCIP in a small group of patients (*n* = 31) to determine the feasibility of implementing the screening tool in our hospital, and to begin to assess the external validity of the German translation of the SCIP [30]. To investigate the external validity of the SCIP, the Zahlen-Verbindungs-Test (ZVT, [31]) was also administered to this patient sample. The ZVT [31] is a non-verbal, simple, standardized and well-established measure of cognitive processing speed. The ZVT can be completed in 4–10 min and it requires subjects to draw lines to connect numbers from 1 to 90 which are positioned more or less randomly on a piece of paper. Pearson correlations of the SCIP values with the age-normalized standard value of the ZVT (mean value 100, standard deviation 10) were calculated.

On average, the *n* = 31 patients in this pilot study were 40.3 years of age (SD = 11.00). The majority of the examined patients (74%) suffered from a psychotic disorder (F2 ICD-10), and only 13% had an affective disorder (F3 ICD-10) as the primary diagnosis. The average administration time for the SCIP was 13.6 min (SD = 2.22). Patients achieved an average total score of 58.55 (SD = 15.67) on the SCIP and a scaled score of M = 85.58 (SD = 11.41) on the ZVT. The SCIP total score correlated significantly with the scaled score from the ZVT (r = .68; *p* < .001). The subscales of the SCIP also correlated significantly between r = .44 and r = .57 with the ZVT scaled score [30]. The relatively brief administration time, high sensitivity to cognitive impairment, and the reasonable validation against the ZVT together supported the feasibility of our intended widespread adoption of the SCIP for our routine inpatient assessments [32].

#### Electronic medical file data

Clinically established primary and secondary diagnoses were extracted from the electronic medical files as well as dates of birth, admission and discharge. Sociodemographic data including the Clinical Global Impression Scale (CGI) were used as recorded by the basic documentation as recommended by the German Association of Psychiatry and Psychotherapy (DGPPN) and validated by Jaeger et al. [33].

### Statistical analysis

All analyses were conducted using IBM SPSS for windows version 24. Some patients were only tested at admission. These patients were compared with patients tested at admission and discharge to investigate a possible selection bias. Chi^2^-Tests were used for normally scaled data, in all other cases we calculated Mann-Whitney-U Tests. To investigate diagnosis-specific differences in SCIP results at admission, a multivariate analysis of variance was calculated for the total value of the SCIP and its subscales, with diagnosis as an independent variable. Variance homogeneity was checked with the Levene test. Pillai-Bartlett trace was used for the test statistics, because it is robust to violations of the multinormal distribution and variance homogeneity [34]. After Bonferroni adjustment, the significance level was set to *p* < .008. For parameter estimation, beta was set to zero for the group of patients with affective disorder (F3 ICD-10). Repeated-Measures ANOVAs were calculated to investigate diagnostic and time effects (admission vs. discharge) as well as interaction effects on the SCIP test values. The test statistics were applied as described above. To facilitate the interpretation of the test results, we standardized the SCIP results using the values reported from a prior healthy sample [2]. By convention we assigned observed scores that were lower than normal by one standard deviation to a pathological classification. Changes in normalized test values over time were analyzed with Chi^2^-Tests. Cohen’s d was calculated as effect sizes. According to Cohen [35], values between 0.2 and 0.5 indicate a small effect, between 0.5 and 0.8 a medium effect and values greater than 0.8 a strong effect. For calculating effect sizes of nominal scaled variables, the Phi coefficient (φ) was used. Here, values above 0.1 are considered to be small effects, values above 0.3 are medium effects and values above 0.5 are large effects [35]. A hierarchical cluster analysis (Ward’s cluster method with squared Euclidean distance measurements) was performed with the SCIP results at admission to identify groups of study subjects with similar cognitive profiles. Afterwards differences between the two clusters were investigated with Chi^2^-Tests and Mann-Whitney-U Tests.

## Results

### Analysis of the admission test scores of the total sample

#### Sample characteristics

The study included data from a total of 529 SCIP results at admission. The patients examined were on average 43.7 years of age (SD = 11.5), male (56.7%), unmarried (60.1%), and without migration background (74.5%). Not quite a third (30%) lived on state support (unemployment benefit, social welfare, sick pay, etc.). The most common primary diagnosis (82.4%) was a psychotic disorder (F2 ICD-10), and only 17.6% (*n* = 93) of the sample were diagnosed with an affective disorder (F3 ICD-10). Of the latter, *n* = 49 patients had a diagnosis of a manic episode or a bipolar affective disorder and *n* = 44 patients had a diagnosis of a depressive episode or a recurrent depressive disorder. On average, patients had 1.77 (SD = 2.00) comorbid diagnoses. 41.6% had more than 5 previous admissions. In the CGI, 46.7% were assessed as “clearly ill” and 24.6% as “seriously ill”. Thus the values in the CGI, the number of pre-treatments and the number of further diagnoses all suggest a relatively high disease severity suffered by the patients examined. The sample characteristics for the total sample are displayed in Table 1.

**Table 1:** Sample characteristics of participants of the total sample (*n* = 529) and the subsample with admission and discharge results (*n* = 227), as well as significant differences between the subsample and drop-outs (without discharge results)

			total sample (admission results)		subsample (admission and discharge results)		drop-outs (only admission results)		p
			*N* = 529		*N* = 227		*N* = 302		
**Sociodemographic characteristics**									
male		n (%)	300	(56.7%)	118	(52.0%)	182	(60.3%)	.057^A^
age (in years)		M (SD)	43.73	(11.53)	45.00	(11.28)	42.77	(11.65)	<.05^B^
unmarried		n (%)	370	(69.9%)	158	(69,6%)	212	(70.3%)	.090^A^
regular employment		n (%)	96	(18.1%)	42	(18.5%)	54	(17.9%)	.854^A^
no migration background		n (%)	394	(74.5%)	180	(79.3%)	214	(70.9%)	<.01^A^
German as native language		n (%)	410	(77.5%)	183	(80.6%)	227	(75.2%)	<.01^A^
**Clinical characteristics**									
primary diagnoses	F2	n (%)	436	(82.4%)	177	(78.0%)	259	(85.8%)	.066^A^
	F3	n (%)	96	(17.6%)	50	(22.0%)	43	(14.2%)	
length of inpatient stay (in days)		M (SD)	46.17	(45.09)	48.44	(40.60)	44.46	(48.20)	<.01^B^
voluntarily admitted		n (%)	377	(71.3%)	159	(70.0%)	218	(72.2%)	.991^A^
no. of previous admissions	1	n (%)	40	(7.6%)	15	(6.6%)	25	(8.3%)	.459^A^
	2–5	n (%)	150	(28.4%)	70	(30.8%)	80	(26.5%)	
	> 5	n (%)	220	(41.6%)	91	(40.1%)	129	(42.7%)	
degree of severity of the disease (CGI)	Borderline case	n (%)	1	(0.2%)	1	(0.4%)	0	(0%)	.495^B^
	only slightly ill	n (%)	8	(1.5%)	1	(0.4%)	7	(2.3%)	
	moderately ill	n (%)	75	(14.2%)	32	(14.1%)	43	(14.2%)	
	clearly ill	n (%)	247	(46.7%)	112	(49.3%)	135	(44.7%)	
	seriously ill	n (%)	130	(24.6%)	51	(22.5%)	79	(26.2%)	
	extremely seriously ill	n (%)	9	(1.7%)	2	(0.9%)	7	(2.3%)	

A: Chi^2^-Test; B: Mann-Whitney-U-Test

Table 1 Sample characteristics of participants of the total sample (*n* = 529) and the subsample with admission and discharge results (*n* = 227), as well as significant differences between the subsample and drop-outs (without discharge results).

#### *Outcome 1:* absolute test values of cognitive performance

The descriptive values of the cognitive performance in SCIP are presented in Table 2. The multivariate model showed no significant diagnosis-specific differences for all SCIP scales (F (5, 523) = 1.568; *p* > .05). Also the post-hoc individual tests showed no significant differences between patients with F2 and F3 diagnosis in the SCIP total score (F (1, 527) = 4.547; *p* > .008) or in SCIP subscale scores (VLT-I: F (1. 527) = 3.240; p > .008; WMT: F (1. 527) = 6.055; p > .008; VFT: F (1. 527) = 0.245; p > .008; VLT-D: F (1. 527) = 3.039; p > .008; PST: F (1. 527) = 3.308; p > .008).

**Table 2:** Mean, standard deviation and percentage of patients with pathological results of cognitive performance at admission for the total sample and separated for ICD-10 F2 and F3 primary diagnosis

	Total sample *N* = 529			F2 *N* = 436			F3 *N* = 93		
	M	(SD)	%Path	M	(SD)	%Path	M	(SD)	%Path
VLT-I	19.84	(5.04)	38.2%	19.65	(5.09)	40.1%	20.69	(4.75)	29.0%
WMT	18.06	(4.59)	54.8%	17.83	(4.66)	57.1%	19.12	(4.11)	44.1%
VFT	13.17	(5.49)	66.2%	13.12	(5.44)	67.7%	13.43	(5.73)	59.1%
VLT-D	5.47	(2.62)	34.2%	5.38	(2.60)	35.1%	5.90	(2.66)	30.1%
PST	8.14	(3.45)	58.4%	8.02	(3.34)	58.9%	8.73	(3.90)	55.9%
Total SCIP	64.69	(15.92)	70.7%	64.01	(15.83)	72.2%	67.87	(16.04)	63.4%

%Path: Percentage of those that are at least 1 standard deviation below the values of [2]; *VLT-I* verbal learning test - immediate; *WMT* working memory test; *VFT* verbal fluency test; *VLT-D* verbal learning test - delayed; *PST* processing speed test; *Total SCIP* SCIP total score; *M* mean; *SD* standard deviation

Table 2 Mean, standard deviation and percentage of patients with pathological results of cognitive performance at admission for the total sample and separated for ICD-10 F2 and F3 primary diagnosis.

#### *Outcome 2:* normalized test values of cognitive performance

Analyzing the rate of impaired SCIP performance in the sample, we found high percentages in the subtests for working memory (WMT), verbal fluency (VFT) and processing speed (PST). In the SCIP total score more than 70% of the patients had impaired performances. Only the cognitive performance in verbal learning was pathological in less than 50% of patients (see Table 2). Corresponding to the absolute SCIP results, there were no diagnosis-specific differences in the normalized values for F2 versus F3 (VLT-I: Chi^2^(1.529) = 5.250; *p* > .008; WMT: Chi^2^(1.529) = 5.250; p > .008; VFT: Chi^2^(1.529) = 2.486; p > .008; VLT-D: Chi^2^(1.529) = 0.846; p > .008; PST: Chi^2^(1. 529) = 2.90; p > .008; Total SCIP: Chi^2^(1.529) = 2.870; p > .008). Descriptive values showed the highest diagnostic differences in the intermediate verbal learning test (40.1% vs. 29.0%) and the working memory test (57.1% vs. 44.1%). In both subscales, the proportion of patients with pathological results was higher in the F2 group than in the F3 group (Table 2).

### Analysis of the subsample with admission and discharge results

#### Sample characteristics

In *n* = 227 patients (42.1%) the SCIP was performed at admission and discharge. A possible selection bias between patients with admission and discharge findings and those without discharge findings (drop-outs) was investigated. Drop-outs differed from patients with discharge results in age (U = 30,288.50; *p* < .05), length of inpatient stay (U = 29,647.00; *p* < .05), native language (Chi^2^ (1)=6.921; *p* < .01) and migration background (Chi^2^ (6)=18.320; *p* < .01). In other variables, especially those related to disease severity (CGI, number of pretreatments), the two groups did not differ (Table 3). Thus, a selection bias is more likely with respect to treatment duration and language barrier than in the area of disease severity (see also Table 1).

**Table 3:** Results of cognitive performance at admission and discharge

	admission		discharge			
	M	(SD)	M	(SD)	p	d
VLT-I	19.83	(5.01)	20.22	(4.74)	n.sign.	0.08
WMT	17.98	(4.38)	18.89	(4.30)	<.008	0.21
VFT	13.26	(5.43)	14.37	(4.84)	<.008	0.22
VLT-D	5.58	(2.57)	5.65	(2.61)	n.sign.	0.03
PST	7.90	(3.43)	9.03	(3.56)	<.001	0.32
Total SCIP	64.55	(15.60)	68.16	(14.67)	<.001	0.24

*VLT-I* verbal learning test - immediate; *WMT* working memory test; *VFT* verbal fluency test; *VLT-D* verbal learning test - delayed; *PST* processing speed test; *Total SCIP* SCIP total score; *N* = 227; p: level of significance; d: effect size

#### *Outcome 1:* absolute test values of cognitive performance

For the 227 patients with admission and discharge testing, the SCIP total score showed a significant time effect (time: F (1.225) = 24.626; *p* < .001), patients improved significantly between admission and discharge. This time effect was also observed in the described direction for the three subscales working memory test (F (1.225) = 8.330; *p* < .008), verbal fluency test (F (1.225) = 10.850; *p* < .008) and processing speed test (F (1.225) = 39.551; *p* < .001). As Table 3 shows, all effects were small. The remaining two subscales showed no significant time effects (VLT-I: F (1.225) = 3.531; *p* > .008; VLT-D: F (1.225) = 1.085; p > .008).

Table 3 Results of cognitive performance at admission and discharge.

#### *Outcome 2:* normalized test values of cognitive performance

**Fig. 1:**
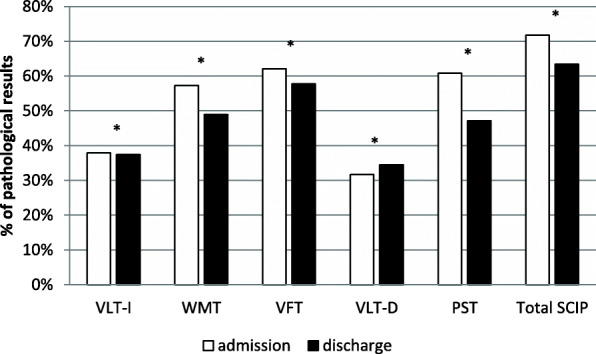
Percentages of pathological results of cognitive performance at admission and discharge in the SCIP total score (Total SCIP) and the five subscales (VLT-I: verbal learning test - immediate; WMT: working memory test; VFT: verbal fluency test; VLT-D: verbal learning test - delayed; PST: processing speed test); *n* = 227; *: *p* < .001

Percentages of the sample with pathological SCIP results at the time of admission and at the time of discharge are presented in Fig. 1. In the SCIP total score (Chi^2^(1, 227) = 87,839; *p* < .001; φ = 0.62) and also in four subscales (VLT-I: Chi^2^(1, 227) = 45,260; *p* < .001; φ = 0.45; WMT: Chi^2^(1, 227) = 43,002; *p*<. 001; φ = 0.44; VFT: Chi^2^(1, 227) = 50,386; *p* < .001; φ = 0.47; PST: Chi^2^(1, 227) = 75,723; *p* < .001; φ = 0.58) the proportion of those impaired decreased significantly over time. Only in the delayed verbal learning test was the proportion of impaired persons greater at discharge than at admission (VLT-D: Chi^2^(1, 227) = 53.078; *p* < .001; φ = − 0.48). Large effect sizes resulted for the SCIP total score (φ = 0.62) and for the processing speed test (φ = 0.58). Otherwise, effects of medium size emerged.

### Outcome 3: identification of groups with different cognitive performance profiles

**Fig. 2:**
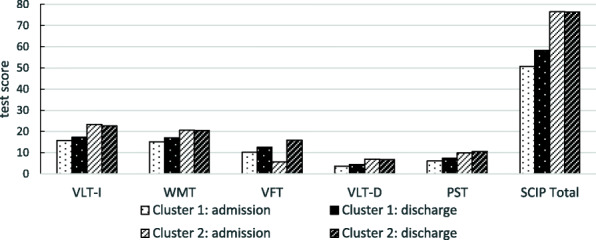
SCIP profiles of the two clusters at admission (*n* = 529) and discharge (*n* = 227) for the SCIP total score (Total SCIP) and the five subscales (VLT-I: verbal learning test - immediate; WMT: working memory test; VFT: verbal fluency test; VLT-D: verbal learning test - delayed; PST: processing speed test)

To assess heterogeneity of cognitive profiles within the clinical sample, we undertook a hierarchical cluster analysis based on the SCIP results at admission. A two cluster solution with *n* = 243 and *n* = 286 patients yielded the best fit. Patients belonging to cluster 1 were significantly older (U = 21,462.00; *p* < .001), had more additional diagnoses (U = 28,328.5; *p* < .001) and had significantly more than 5 previous admissions (Chi^2^ (2)=13.375; *p* < .001) compared to patients belonging to cluster 2. In the SCIP admission scores, patients of the first cluster scored significantly worse in all test results compared to patients of cluster 2 (see Table 4, see also Fig. 2). All patients of cluster 1 showed pathological SCIP total scores at admission, whereas only 45,8% of the patients of cluster 2 show (Chi^2^(1529) = 186,28; *p* < .001).

**Table 4:** Differences in sociodemographic variables and SCIP profiles between the two clusters based on SCIP results at admission

total sample			Cluster		Cluster 2		
			n = 243		n = 286		p
male		n (%)	147	(60.5%)	153	(53.5%)	n.sign.^A^
age		M (SD)	47.88	(10.27)	40.20	(11.39)	<.001^B^
length of inpatient stay		M (SD)	46.09	(45.23)	46.23	(44.98)	n.sign.^B^
F2 as primary diagnoses		n (%)	212	(87.2%)	224	(78.3%)	n.sign.^A^
no. of comorbid diag.		M (SD)	2.12	(2.18)	1.47	(1.80)	<.001^B^
no. of pre-treatments	1	n (%)	12	(2,9%)	28	(6,8%)	<.001^A^
	2–5	n (%)	62	(31,5%)	88	(41,3%)	
	> 5	n (%)	123	(62,4%)	97	(45,5%)	
admission	VLT-I	M (SD)	15.70	(3.59)	23.35	(3.03)	<.001^B^
	WMT	M (SD)	15.08	(4.14)	20.59	(3.23)	<.001^B^
	VFT	M (SD)	10.16	(4.41)	15.73	(5.00)	<.001^B^
	VLT-D	M (SD)	3.69	(2.09)	6.99	(1.99)	<.001^B^
	PST	M (SD)	6.12	(2.39)	9.86	(3.29)	<.001^B^
	SCIP Total	M (SD)	50.75	(9.78)	76.53	(9.05)	<.001^B^
	Pathol. SCIP Total	n (%)	243	(100,0%)	131	(45,8%)	<.001^A^
**subsample**		N (%)	106	(43.8%)	121	(42.2%)	
discharge	VLT-I	M (SD)	17.26	(4.18)	22.72	(3.63)	<.001^B^
	WMT	M (SD)	17.07	(4.26)	20.44	(3.70)	<.001^B^
	VFT	M (SD)	12.52	(4.64)	15.94	(4.46)	<.001^B^
	VLT-D	M (SD)	4.37	(2.33)	6.77	(2.32)	<.001^B^
	PST	M (SD)	7.33	(2.75)	10.46	(3.54)	<.001^B^
	SCIP Total	M (SD)	58.37	(12.68)	76.45	(10.59)	<.001^B^
	Pathol. SCIP Total	n (%)	94	(90,4%)	50	(40,7%)	<.001^A^
improvement	VLT-I	M (SD)	1.57	(4.46)	−0.61	(3.97)	<.001^B^
	WMT	M (SD)	1.82	(4.06)	0.15	(3.41)	n.sign ^B^
	VFT	M (SD)	2.15	(4.30)	0.24	(4.29)	n.sign ^B^
	VLT-D	M (SD)	0.38	(2.18)	−0.20	(2.24)	n.sign.^B^
	PST	M (SD)	1.41	(2.12)	0.89	(2.75)	n.sign.^B^
	SCIP Total	M (SD)	7.34	(10.64)	0.46	(9.12)	<.001^B^

*VLT-I* verbal learning test - immediate; *WMT* working memory test; *VFT* verbal fluency test; *VLT-D* verbal learning test - delayed; *PST* processing speed test; *Total SCIP* SCIP total score; A: Chi^2^-Test; B: Mann-Whitney-U-Test; improvement = discharge - admission

To make statements about possible improvements in cognitive performance during treatment, the subsample with admission and discharge results was analyzed. For those *n* = 106 and *n* = 121 patients the discharge results and the improvement between admission and discharge are presented in Table 4. As with the findings at admission, the discharge results showed that cluster 1 had significantly worse results in all scales than cluster 2. In terms of improvement, patients in the first cluster scored significantly higher in the SCIP total score and in the immediate verbal learning test compared to patients in the second cluster.

Table 4 Differences in sociodemographic variables and SCIP profiles between the two clusters based on SCIP results at admission.

## Discussion

The SCIP is an economical, internationally approved and validated screening tool to assess cognitive performance in psychiatric patients. In this report we provide data of a naturalistic sample of 529 psychiatric inpatients screened with the SCIP at the time of admission to a general psychiatric ward. This is the first paper to report data of such a sample. Results for the SCIP total score and the five subscale scores corresponded to published data from other translations of the SCIP (e.g. [2]). Diagnosis-specific differences between psychotic and affective disorders in our sample did not occur. Our results are therefore consistent with the results of Gómez-Benito et al. [8], Sachs et al. [11], Neu et al. [10] and Hill et al. [9]. In addition to the absolute values, we used the results of the healthy population norms from Murri et al. [2] to investigate the proportion of our sample with pathological results. This revealed a high percentage of cognitive dysfunction in our sample evident in the SCIP total score (over 70%) as well as for the three subtests with significant demands for attention and processing components (working memory, verbal fluency test, psychomotor speed test). The subtests of verbal learning and memory on the other hand were pathological in less than 40% of the patients.

Looking at the cognitive performance profiles over the course of inpatient treatment, there were significant improvements from admission to discharge in the total score and the subtests for verbal fluency, processing speed and working memory. In contrast, verbal learning, both immediate and delayed, showed no changes over time. This is in contrast to previous findings with reported deficits as well as in the domains of verbal memory and processing speed [3, 4]. In terms of the normalized values, large effect sizes were observed for the improvement in the SCIP total score and the psychomotor speed test between admission and discharge. Taken together, most cognitive improvements occurred in working memory, processing speed and verbal fluency (which also includes a speed factor). We suppose that social psychiatric inpatient treatment improves most in cognitive domains of attention, working memory and speed components, because in daily ward living, social interaction and communication are key processes within the recovery model. Possible effects of the reapplied psychiatric medication during an inpatient stay must also be taken into account. On the one hand, improvements in cognitive performance are conceivable, particularly if second generation antipsychotics are given [36, 37] on the other hand, side effects such as fatigue and loss of attention could also occur [10]. In the present study, the influence of medication effects could not be analyzed.

Following Rodriguez et al. [38] we further investigated whether different patient clusters could be identified based on the results of a screening tool. In our analysis, two clusters were extracted based on SCIP results at admission. Rodriguez et al. [38] extracted three clusters corresponding to a general impairment group, an attention, speed and verbal deficit group, and a third cluster with above average attention but impaired verbal memory. In contrast to Rodriguez et al. [38], the sample reported here separated into either one group with mild impairment in terms of mean value with almost 50% of pathological results on the SCIP (cluster 2) and 1 sec group with severe impairment in terms of mean with 100% of pathological results on the SCIP (cluster 1). Patients of cluster 1 were older, had more comorbid diagnoses and had more previous admissions. However, compared to cluster 2, they improved significantly more between admission and discharge results, both in the overall score and in the verbal learning test. Since the patients of cluster 2 are already considerably less impaired in the admission findings based on the values of Murri et al. [2], a ceiling effect must be considered for this cluster. In conclusion, cluster 1 could be interpreted as a sample of chronic patients, more impaired clinically as well as cognitively, who then benefitted from the social psychiatric inpatient stay.

Our study has several limitations. First, our results are based on routinely collected clinical data by a screening tool for cognitive performance. Beside the ecological validity advantages of such a naturalistic clinical dataset, selection processes of the examined patients must be discussed. Only those, who were willing to participate in such a diagnostic procedure, could be included. To investigate possible selection processes concerning repeated measurement, drop-out analyses were performed. Compared to the complete cases with both admission and discharge data, the patients that dropped out prior to the discharge evaluation differed only in sociodemographic variables, and a shorter length of stay. It can therefore be assumed that selection was based more on organizational concerns and less on disease severity. Furthermore it must be noted that patients with cognitive impairment due to other disorders are regularly not treated on the units where the patients were recruited but presence of such conditions in a milder degree had not been predefined as an exclusion criterion. Comorbid disorders should be considered more in future work.

Another limiting factor is the lack of a concurrent healthy control sample for comparison, and the absence of published data with reported SCIP scores from a German translation of the SCIP. We selected the relatively large sample reported by Murri et al. [2] from the Italian translation over similar reports from the Spanish translation [25, 26]. When comparing with the healthy sample of Murri et al. [2], the differences in age, gender distribution and educational status compared to our sample must be mentioned as a limitation. With the current demonstration of the sensitivity of the German translation of the SCIP to the cognitive limitations apparent in an inpatient psychiatric sample we are confident that the time and effort required to secure normative data for this version would be very well spent. As in all investigations of cognitive limitations, we were not able to include in our analysis many additional factors that could influence or potentially mediate test results, e.g. medication or learning effects. Moritz et al. [39] were able to show that factors such as motivation and attitude towards testing or medication significantly reduce cognitive abnormalities in schizophrenia sufferers. This all should be considered in future work.

## Conclusion

In conclusion, the SCIP has proven to be a suitable instrument for routine use in the detection of cognitive impairment. This is particularly important because of the impact of cognition for psychosocial functioning. Our results further indicate an improvement in cognitive performance during an inpatient stay. This may be particularly relevant to chronically ill patients with many previous admissions because we might anticipate substantial gains during the admission that could be communicated back to the patient and their family to instill some hope for even partial recovery.

## Data Availability

The datasets used and analysed during the current study are available from the corresponding author on reasonable request.

## References

[1] Kraepelin E (1899). Psychiatrie. Ein Lehrbuch für Studierende und Aerzte. 6. Aufl., Leipzig.

[2] Murri MB, Folesani F, Costa S, Biancosino B, Colla C, Zerbinati L, Caruso R, Nanni MG, Purdon SE, Grassi L. Screening for cognitive impairment in non-affective psychoses: A comparison between the SCIP and the MoCA. Schizophrenia Res 2020; 218: 188–194. doi.org/10.1016/j.schres.2020.01.005 10.1016/j.schres.2020.01.005 31948897

[3] Mesholam-Gately RI, Giuliano AJ, Go KP, Faraone SV, Seidman LJ. Neurocognition in first-episode schizophrenia: a meta-analytic review. Neuropsychology 2009; 23: 315–336. doi.org/10.1037/a0014708 10.1037/a0014708 19413446

[4] Zanelli J, Mollon J, Sandin S, Morgan C, Dazzan P, Pilecka I, et al. Cognitive change in schizophrenia and other psychoses in the decade following the first episode. Am J Psychiatry. 2019:appiajp201918091088. 10.1176/appi.ajp.2019.18091088. 10.1176/appi.ajp.2019.18091088 31256609

[5] Dickinson T, Becerra R, Coombes J (2017). Executive functioning deficits among adults with bipolar disorder (types I and II): a systematic review and meta-analysis. J Affect Disord.

[6] Rock PL, Roiser JP, Riedel WJ, Blackwell AD (2014). Cognitive impairment in depression: a systematic review and meta-analysis. Psychol Med.

[7] Ahern E, Semkovska M (2017). Cognitive functioning in the first-episode of major depressive disorder: a systematic review and meta-analysis. Neuropsychology..

[8] Gómez-Benito J, Guilera G, Pino Ó, Rojo E, Tabarés-Seisdedos R, Safont G, Martínez-Arán A, Franco M, Cuesta MJ, Crespo-Facorro B, Bernardo M, Vieta E, Purdon SE, Mesa F, Rejas J, the Spanish Working Group in Cognitive Function (2013). The screen for cognitive impairment in psychiatry: diagnostic-specific standardization in psychiatric ill patients. BMC Psychiatry.

[9] Hill SK, Reilly JL, Harris MS, Rosen C, Marvin RW, Deleon O, Sweeney JA (2009). A comparison of neuropsychological dysfunction in first-episode psychosis patients with unipolar depression, bipolar disorder, and schizophrenia. Schizophr Res.

[10] Neu P, Gooren T, Niebuhr U, Schlattmann P (2019). Cognitive impairment in schizophrenia and depression: a comparison of stability and course. Appl Neuropsychol Adult.

[11] Sachs G, Maihofer E, Erfurth A. Are deficits in social cognition differentiating between schizophrenia and affective disorders. Eur Psychiatry 2017; 41 Sup: S44–S45. DOI: doi.org/10.1016/j.eurpsy.2017.01.196

[12] Bonner-Jackson A, Grossman LS, Harrow M, Rosen C. Neurocognition in schizophrenia: a 20-yearmulti-follow-upofthecourseofprocessing speed and stored knowledge. Compr Psychiatry 2010; doi.org/10.1007/s00406-019-01030-z, 2020. 10.1016/j.comppsych.2010.02.005 PMC2927366 20728003

[13] Wu C, Dagg P, Molgat C (2017). Measuring stability of cognitive impairment in inpatients with schizophrenia with alternate forms of the Montreal cognitive assessment during acute hospitalization. Psychiatry Res.

[14] Krug A, Stein F, Kircher T. Kognitive Störungen bei Schizophrenie. Cognitive disorders in schizophrenia. Der Nervenarzt. 2020;91(1):2–9. 10.1007/s00115-019-00809-8. 10.1007/s00115-019-00809-8 31559478

[15] Christensen TØ. The influence of neurocognitive dysfunctions on work capacity in schizophrenia patients: a systematic review of the literature. Int J Psychiatry Clin Pract 2007; 11: 89–101. doi.org/10.1080/13651500600969061 10.1080/13651500600969061 24937554

[16] Malla AK, Norman RMG, Manchanda R, Townsend L. Symptoms, cognition, treatment adherence and functional outcome in first-episode psychosis. Psychol Med 2002; 32: 1109–1119. doi.org/10.1017/S0033291702006050 10.1017/s0033291702006050 12214790

[17] Tourjman SV, Juster R-P, Purdon S, Stip E, Kouassi E, Potvin S (2018). The screen for cognitive impairment in psychiatry (SCIP) is associated with disease severity and cognitive complaints in major depression. Int J Psychiatry Clin Pract.

[18] Caletti E, Paoli RA, Fiorentini A, Cigliobianco M, Zugno E, Serati M, Orsenigo G, Grillo P, Zago S, Caldiroli A, Prunas C, Giusti F, Consonni D, Altamura AC (2013). Neuropsychology, social cognition and global functioning among bipolar, schizophrenic patients and healthy controls: preliminary data. Front Hum Neurosci.

[19] Knight MJ, Air T, Baune BT (2018). The role of cognitive impairment in psychosocial functioning in remitted depression. J Affect Disord.

[20] Medalia A, Saperstein AM (2013). Does cognitive remediation for schizophrenia improve functional outcomes?. Curr Opin Psychiatry.

[21] Purdon SE (2005). The screen for cognitive impairment in psychiatry (SCIP): instructions and three alternate forms.

[22] Keefe RSA, Goldberg TE, Harvey PD, Gold JM, Poe MP, Coughenour L. The brief assessement of cognition in schizophrenia: reliability, sensitivity, and comparison with standard neurocognitive battery. Schizophr Res 2004; 68: 283-297.doi.org/10.1016/j.schres.2003.09.011 10.1016/j.schres.2003.09.011 15099610

[23] Nasreddine Z. The Montreal cognitive assessment, MoCA: a brief screening tool for mild cognitive impairment. J Am Geriatr Soc 2005; 695–699. doi.org/10.1111/j.1532-5415.2005.53221.x 10.1111/j.1532-5415.2005.53221.x 15817019

[24] Cuesta MJ, Pino O, Guilera G, Rojo JE, Gómez-Benito J, Purdon SE, Franco M, Martínez-Arán A, Segarra N, Tabarés-Seisdedos R, Vieta E, Bernardo M, Crespo-Facorro B, Mesa F, Rejas J (2011). Brief cognitive assessment instruments in schizophrenia and bipolar patients, and healthy control subjects: a comparison study between the brief cognitive assessment tool schizophrenia (B-CATS) and the screen for cognitive impairment in psychiatry (SCIP). Schizophr Res.

[25] Guilera G, Pino O, Gómez-Benito J (2009). Clinical usefulness of the screen for cognitive impairment in psychiatry (SCIP-S) scale in patients with type I bipolar disorder. Health Qual Life Outcomes.

[26] Pino O, Guilera G, Rojo JE, Gómez-Benito J. et al. Spanish version of the Screen for Cognitive Impairment in Psychiatry (SCIP-S): psychometric properties of a brief scale for cognitive evaluation in schizophrenia. Schizophr Res. 2008; 99(1–3):b139–b148. doi.org/10.1016/j.schres.2007.09.012 10.1016/j.schres.2007.09.012 17959358

[27] Rojo E, Pino O, Guilera G, Gómez-Benito J, Purdon SE, Crespo-Facorro B, Cuesta MJ, Franco M, Martínez-Arán A, Segarra N, Tabarés-Seisdedos R, Vieta E, Bernardo M, Mesa F, Rejas J, Spanish Working Group in Cognitive Function (2010). Neurocognitive diagnosis and cut-off scores of the screen for cognitive impairment in psychiatry (SCIP-S). Schizophr Res.

[28] Ott CV, Bjertruo AJ, Jensen JH, Ullum H et al. Screening for cognitive dysfunction in unipolar depression: validation and evaluation of objective and subjective tools. J Affect Disord 2016; 190: 607–615. doi.org/10.1016/j.jad.2015.10.059 10.1016/j.jad.2015.10.059 26583350

[29] Tourjman SV, Beauchamp MH, Djouini A, Neugot-Cerioli M, Gagner C, Baruch P, Beaulieu S, Chanut F, Daigneault A, Juster RP, Montmayeur S, Potvin S, Purdon S, Renaud S, Villenneuve E (2016). French validation of the screen for cognitive impairment in psychiatry (SCIP-F). Open J Psychiatry.

[30] Czekaj A, Uhlmann C, Flammer E, Frick J. et al. Klinische Praktikabilität der "Erfassung kognitiver Beeinträchtigung" bei Patienten der Allgemeinpsychiatrie (Screen for Cognitive Impairment in Psychiatry/SCIP). Posterpräsentation, DGPPN, Berlin, 21.-24.11.2012.

[31] Oswald WD, Roth R (1987). Zahlen-Verbindungs-Test (ZVT).

[32] Czekaj A, Steinert T, Uhlmann C (2014). Klinische Routinediagnostik kognitiver Beeinträchtigungen. Psychiatr Prax.

[33] Jaeger S, Flammer E, Steinert T (2011). Basisdokumentation in der klinischen Praxis: Wie zuverlässig sind BADO-Daten?. Psychiatr Prax.

[34] Field A. Discovering Statistics using SPSS. London: Sage Publications Ltd; 2005.

[35] Cohen J. Statistical power analysis for the behavioral sciences. 2nd ed. 1988. https://doi.org/10.1234/12345678.

[36] Purdon SE, Jones BD, Stip E, Labelle A, Addington D, David SR, Breier A, Tollefson GD (2000). Neuropsychological change in early phase schizophrenia during 12 months of treatment with olanzapine, risperidone, or haloperidol. The Canadian collaborative group for research in schizophrenia. Arch Gen Psychiatry.

[37] Woodward ND, Purdon SE, Meltzer HY, Zald DH (2005). A meta-analysis of neuropsychological change to clozapine, olanzapine, quetiapine, and risperidone in schizophrenia. Int J Neuropsychopharmacol.

[38] Rodriguez M, Zaytseva Y, Cvrčková A (2019). Cognitive profiles and functional connectivity in first-episode schizophrenia Spectrum disorders - linking behavioral and neuronal data. Front Psychol.

[39] Moritz S, Klein JP, Desler T, Lill H, Gallinat J, Schneider BC (2017). Neurocognitive deficits in schizophrenia. Are we making mountains out of molehills?. Psychol Med.

